# Predation of Cyclopoid Copepods on the Theronts of *Ichthyophthirius multifiliis*: Shedding Light on Biocontrol of White Spot Disease

**DOI:** 10.3390/pathogens12070860

**Published:** 2023-06-22

**Authors:** Ze-Yi Cao, Bing-Wen Xi, Qing-Jie Zhou, Kai Chen, Jun Xie

**Affiliations:** 1 Wuxi Fisheries College, Nanjing Agricultural University, Wuxi 214081, China; caozy@stu.njau.edu.cn (Z.-Y.C.);; 2 Key Laboratory of Aquatic Animal Nutrition and Health, Freshwater Fisheries Research Center, Chinese Academy of Fishery Science, Wuxi 214081, China

**Keywords:** *Ichthyophthirius multifiliis*, theront, copepods, biocontrol

## Abstract

White spot disease, caused by the parasitic ciliate *Ichthyophthirius multifiliis*, is a significant threat to the freshwater fish farming industry worldwide, resulting in massive mortality and economic losses. Eliminating the free-swimming theronts from the culture environment is considered crucial for the control of *I. multifiliis* infection. It is well-documented that planktonic ciliates are valuable food resources for macro-zooplankton in aquatic ecosystems. In this study, we developed a fluorescence labeling method for alive theronts and found that cyclopoid copepods *Thermocyclops taihokuensis*, *Mesocyclops* spp., *Macrocyclops* sp., and *Paracyclopina* sp. present predation on the theronts in co-culture experiments. Laboratory challenge tests further confirmed that the presence of zooplankton in the culture water body significantly reduced the infection of *I. multifiliis* in goldfish (*p* < 0.01). Results from this study revealed that cyclopoid copepods have the potential to be used as biological control agents against white spot disease in aquaculture.

## 1. Introduction

The ciliated protozoan *Ichthyophthirius multifiliis* infests most freshwater fish, causing significant economic losses in aquaculture worldwide, including the ornamental fish [1]. The parasite invades the gills, skin, and fins of fish and establishes parasitism in the epithelium. Mature parasites cause hyperplasia of epithelial tissue, which was macroscopically visible as 0.5–1.0 mm white spots [2]. Severe infection by *I. multifiliis* results in numerous white spots and significantly affects the respiration and osmoregulation of fish hosts, leading to massive mortality [3].

Various chemical and physical interventions have been employed against white spot disease (ichthyophthiriasis) [2,4,5]. Historically, malachite green, mercurous acetate and their derivatives were used to control *I. multifiliis* due to the high efficacy against both the free-swimming stage (tomont and theront) of its life cycle in water and the parasitic stage (trophont) in fish host epithelium [4,6]. However, the carcinogenic and teratogenic effects of these chemicals led to bans on their use in aquaculture. On farms, formalin, copper sulphate, peracetic acid, hydrogen peroxide, sodium percarbonate, and plant extracts (*Zingiber officinale* and *Capsicum annuum*) are used to treat this ciliate disease. The treatments aim to eliminate the infection primarily by targeting the free-living tomonts and theronts. Repetitive use of chemicals is necessary to prevent the continuation of infection. However, repeated and prolonged treatments weaken and stress fish, increase the susceptibility to secondary bacterial infection, and may have negative environmental side-effects [7]. Meanwhile, these treatments face challenges in open aquaculture environments, such as cage-farming in rivers or reservoirs. There is an urgent need to discover novel, effective, and environmentally friendly methods for white spot disease.

In freshwater fish farming, fish in outdoor earthen ponds with abundant plankton are not susceptible to white spot disease, but those in indoor culture systems with clean water are more vulnerable. What is the potential biological mechanism behind this phenomenon? The life cycle of *I. multifiliis* involves three mainly different development stages: trophont, tomont, and theront [1,3]. Theronts are pelagic and highly motile ciliates free-swimming in water with a body size of 20–50 μm. They seek out their fish host in the water after being released from the tomonts [3,6]. In aquatic ecosystems, ciliates play an essential role in the microbial food web, effectively utilizing the production of bacteria and phytoplankton and transferring the energy and materials to larger zooplankton, such as copepods, cladocerans and rotifers [8,9,10,11]. Especially, the trophic link between ciliates and copepods has been well-documented in both marine and freshwater environments [9,10]. 

The predation of larger zooplankton on ciliates raises the hypothesis that free-swimming theronts of *I. multifiliis* could also be predated by copepods, cladocerans, or rotifers in aquatic ecosystems. According to the author’s knowledge, until now there was no information available about zooplankton predation on *I. multifiliis*. In this report, we conducted a series of indoor studies to identify the native predator of theronts by using a fluorescent tracer, and preliminarily studied the effect of larger zooplankton on the infection intensity of *I. multifiliis* in fish.

## 2. Materials and Methods

### 2.1. Ichthyophthirius multifiliis Culture and Isolation

*Ichthyophthirius multifiliis* isolates were collected from goldfish purchased from a pet market in Wuxi, China. Following the methods described by Li et al. [12], an indoor recirculating water system was used to maintain *I. multifiliis* and fish host in a 100 L aquarium under water temperature 23 ± 1 °C, pH 7.1 ± 0.3, DO 5.0–7.0 mg/L. Naïve juvenile gibel carps (*Carassius auratus gibelio*) weighing 30–50 g were obtained from the experimental station of Freshwater Fisheries Research Center (FFRC), and used for maintaining *I. multifiliis* in vivo. Theronts of *I. multifiliis* were prepared following the method described by Clayton and Price [13]. Briefly, fish with visible white spots were immersed in aerated tap water in a 500 mL beaker and left for the trophonts to exit the fish and form tomonts. Tomonts from the bottom of the beaker were collected and transferred to a culture dish containing distilled water. After rinsing three times with distilled water to remove fish mucus, they were incubated at 23.5 ± 0.5 °C for 18–20 h. To determine the concentration of theronts, ten 2 μL droplets of the theront suspension were counted under a microscope, respectively. 

### 2.2. Macro-zooplankton Collection and Identification

Macro-zooplankton samples were collected using a plankton net (64 μm) and divided into two parts: one was fixed immediately in neutral 1% Lugol’s solution (Yuanye, Shanghai, China), and the other was transferred to the lab with pond water for the following co-culture predation experiments. Farmed fish in the ponds were also collected and checked for *I. multifiliis* infection with a microscope. The date and location of each sample are listed in Table 1. The fixed samples were used for morphological identification of zooplankton species composition with the kind help of Professor Li Wu (School of Life Science, Hefei Normal University).

### 2.3. Fluorescent Labeling of Infective Theronts 

5-(and 6)-carboxyfluorescein diacetate succinimidyl ester (CFDA-SE) is widely used as a cell stain in vivo for cell tracking and proliferation studies [14]. Based on the preliminary study, CFDA-SE was chosen and used to label and track the infective theronts of *I. multifiliis* in the following studies. 

CFDA-SE solution (Bestbio, Shanghai, China) was added into the culture dish containing tomonts to a final concentration of 1 μM, and then the culture dish was kept in the dark at 23 °C for 3 h. A 40 μm cell strainer was used to isolate the tomonts from the staining solution. Tomonts were transferred to a new culture dish with distilled water and incubated at 23.5 ± 0.5 °C for 18–20 h. Analysis of theront labeling with CFDA-SE was performed using a fluorescence stereomicroscope (Nikon SMZ18, Tokyo, Japan). As a control, the culture solution without theronts was isolated using a 0.4 μm suction filtration. 

### 2.4. Co-Culture and Predation Experiments

Approximately 2000 macro-zooplankton individuals and 10,000 fluorescence-labeled theronts were added in a plastic container with 1 L dechlorinated tap water. Containers with the same amount of macro-zooplankton and culture solution but without theronts served as controls. After 4 h co-culture under room temperature (23 ± 1 °C), macro-zooplankton were collected with cell strainers (100 μm) and observed under a fluorescence stereomicroscope. To reduce the swimming and jumping of zooplankton under microscopic view, several drops of alcohol were added to the dishes. Zooplankton individuals with fluorescent signals were handpicked individually using pipettes and stored in 95% alcohol. For each macro-zooplankton sample, the co-culture and predation trial was conducted in triplicate. Twenty positive individuals from each sample were randomly selected and coded. They were morphologically identified according to the description by Shen [15] and further confirmed with DNA identification. 

### 2.5. Molecular Analyses

In total, 180 zooplankton individuals were collected and transferred to 0.2 mL PCR tubes, respectively. Genomic DNA was extracted using a lysis buffer for microorganism to direct PCR (TaKaRa, Dalian, China). According to the previous studies, the part of 28s rDNA was amplified with primers CopF2 and CopR1 [16]. PCR reaction contained 25 μL 2 × Taq Master Mix (Dye Plus) (Vazyme, Nanjing, China), 2 μL of each primer (10 μM), 4 μL DNA template and 17 μL ddH_2_O. The following thermocycler conditions were employed: 94 °C for 60 s; 35 cycles of 94 °C for 5 s, 61 °C for 20 s, and 72 °C for 30 s; and a final 72 °C extension step of 10 min. PCR products were purified and sequenced at Sangon Biotech (Shanghai, China). The obtained DNA sequencing chromatograms were checked in Chromas 2.6.6, and sequences were assembled with SeqMan (LaserGene package), aligned in Geneious [17]. Sequence similarity was searched against the GenBank database using the Basic Local Alignment Search Tool (BLAST), and the closest hit with a species identity was recruited. In phylogenetic analyses, 19 copepod sequences and *Ceriodaphnia pulchella* (DQ470627) retrieved from GenBank were aligned with sequences obtained in this study in MAFFT [18]. The phylogenetic relationship was inferred using the maximum likelihood (ML) method in IQ-TREE v1.6.12 [19]. 

### 2.6. Challenge and Infection Level Determination

Goldfish (*Carassius auratus*) weighing 4.2 ± 1.3 g were bought from a local fish market and acclimated for 2 weeks in a 100 L glass aquarium (water temperature 19.5–22.0 °C, pH 7.1 ± 0.3, DO 5.0–7.0 mg/L). Microscopic exams and PCR tests following the previous study [20] were carried out to ensure these fish did not carry *I. multifiliis*.

Macro-zooplankton were collected from an earthen pond in the experimental station of FFRC, and adjusted to a concentration of approximately 1000 ind./L. The zooplankton species compositions were counted under a microscope and identified as copepods, cladocerans, and rotifers (5:3:6). 

One hundred eighty goldfish were divided into six groups with three replicates (Table 2), and the challenge test was conducted in two water sources, tap water (T) and pond water (P). Group T was set as null control, and fish were reared in plastic containers with 2 L aerated tap water without zooplankton and theronts. In group T+I, fish were reared in 2 L aerated tap water with theronts (final concentration, 50,000 cells). In group T+I+Z, fish were reared in tap water with zooplankton (1000 ind./L) and theronts (50,000 cells). Meanwhile, in group P, fish were reared in pond water filtrated with a plankton net (64 μm) set as control (without zooplankton). In group P+I, fish were reared in filtrated pond water (without zooplankton) with theronts. In group P+I+Z, fish were reared in pond water (with zooplankton) and theronts (Table 2). Four days later, fish were anesthetized with MS-222 and dissected. Trophonts on the first gill branch from the left side were counted under the microscope.

### 2.7. Statistical Analysis

All infection intensity data were expressed as mean ± sem and analyzed using one-way analysis of variance (ANOVA) with the Duncan test. The differences were considered significant at *p* < 0.01. Statistical analyses were performed in SPSS 23, and the graph was generated using OriginPro. 

## 3. Results

### 3.1. Theronts Labeled with CFDA-SE

**Figure 1:**
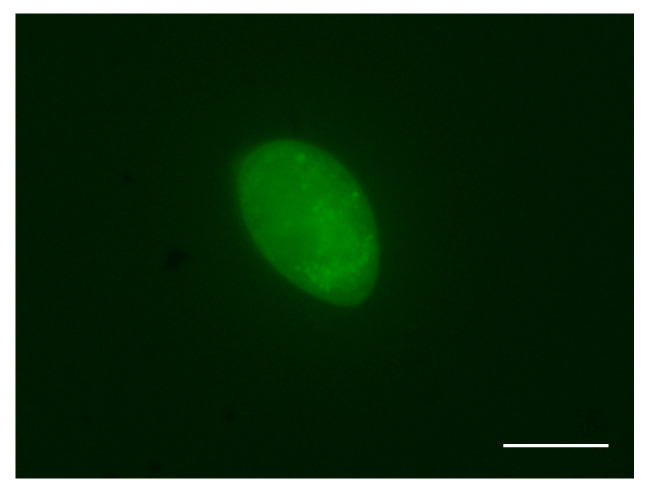
*Ichthyophthirius multifiliis* theronts with a fluorescence label. Scale bar, 20 μm.

After 18 h of incubation, 90% of tomonts successfully released free-swimming theronts, and the theronts emerged from tomont cysts presenting a 100% fluorescent signal under microscopy (Figure 1). The viability of fluorescence-labeled theronts was similar to that of those not treated with CFDA-SE solution, and the fluorescent signal in theronts could persist over 6 h.

### 3.2. Predation of Copepods on Theronts

**Figure 2:**
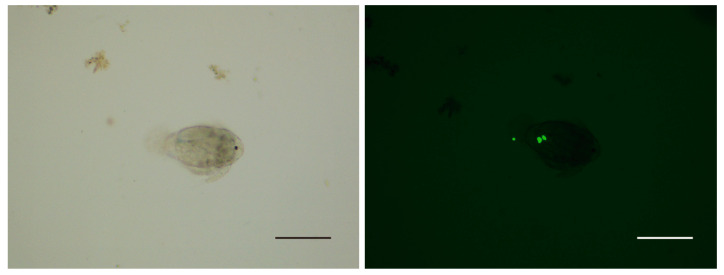
Nauplius with fluorescence-labeled theronts. Scale bars, 200 μm.

**Figure 3:**
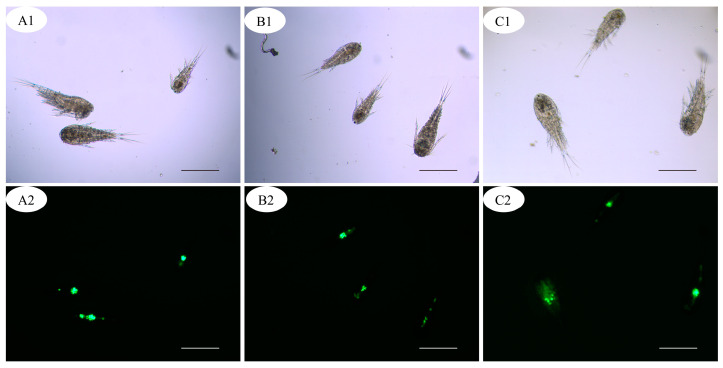
(**A1**–**C2**) Copepods with fluorescence-labeled theronts. Scale bars, 500 μm.

After 4 h co-culture, no fluorescent signal was detected from the zooplankton in the control group. In the group of fluorescence-labeled theronts, the fluorescence signal was mainly observed in copepods and few nauplii (Figure 2 and Figure 3); however, no signal was detected in cladocerans and rotifers. In the alimentary canal of copepods, several fluorescent points, similar in size to fluorescence-labeled theronts, were observed (Figure 3). 

### 3.3. Species of Zooplankton and Predators of Theronts 

Zooplankton collected from six fish ponds and three wild water bodies had high species diversity and consisted of 11–18 species of copepods, cladocerans and rotifers (Table S1). Species compositions in the healthy fish ponds (NQ1, NQ2, NQ3), wild water bodies (LK1, LK2, LK3), and diseased ponds with *I. multifiliis* infection (YX1, YX2, YX3) were different. 

**Figure 4:**
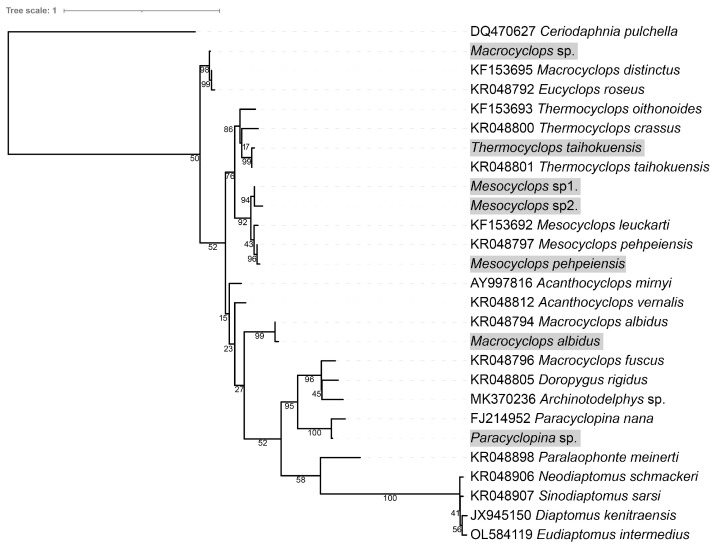
Phylogenetic tree of particular 28s rDNA of copepods. Ultrafast bootstrap support values are shown near the nodes. The species with gray shading are copepods, which prey on theronts in this study.

Co-culture and predation experiments revealed that mature copepods were the main predators of free-swimming theronts of *I. multifiliis*, and only one copepod nauplii individual was observed with a fluorescence signal. The 28s rDNA sequences of 180 copepod individuals were amplified in this study; however, 96 (53.3%) were successfully obtained. Based on morphological characters and DNA sequence analysis, the copepod predators were identified as seven species or operational taxonomic units (OTUs) of the order Cyclopoida, including *Thermocyclops taihokuensis*, *Mesocyclops* sp1., *Mesocyclops* sp2., *Paracvclopina* sp., and *Macrocyclops* sp. (Table 3 and Figure 4). In the identified copepods, *Thermocyclops taihokuensis* and *Mesocyclops* sp1. were the dominant predators, comprising 62.5% (60/96) and 28.1% (27/96), respectively

### 3.4. Intervention Effect of Macro-zooplankton on I. multifiliis Infection in Goldfish

**Figure 5:**
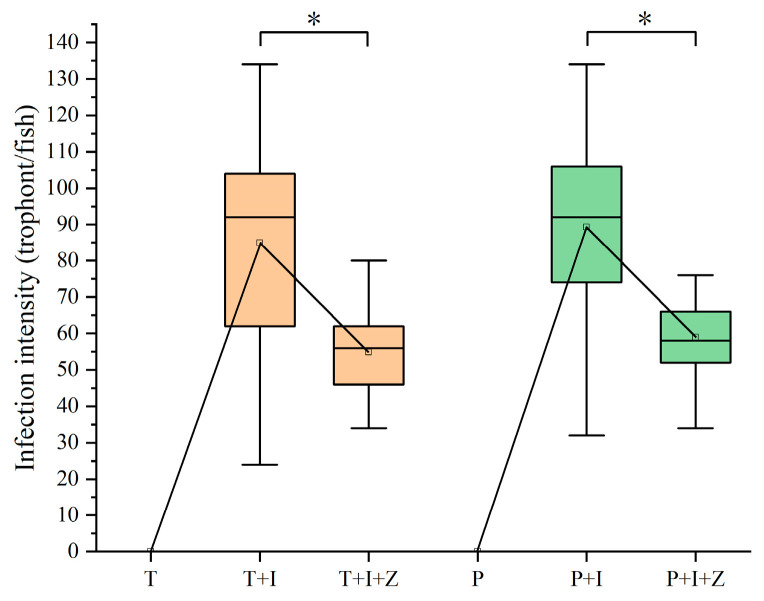
Infection intensity changes of different treatments of goldfish post *I. multifiliis* infection. Significant differences (*p* < 0.01) are denoted by an asterisk (*).

Copepods grazing on the free-swimming theronts would decrease the abundance of theronts in water bodies and reduce the infection pressure of fish hosts. The authors here tried to assess the intervention effect of predators on *I. multifiliis* infection in goldfish using the macro-zooplankton collected in a fish pond (Table 2). The results indicated that predation of copepods on theronts significantly reduced the parasite burden on fish gills (*p* < 0.01) (Figure 5). In tap water, 84.93 ± 5.08 trophonts were detected on the first branch of the left gill in the group T+I (Figure 5); however, only 54.87 ± 2.31 trophonts were observed in the group T+I+Z with zooplankton. Similar results (*p* < 0.01) were seen in the groups raised in pond water (89.27 ± 4.61 to 58.93 ± 1.68). In control groups (T and P), no *I. multifiliis* was found in goldfish, and the water source had no influence on infection by *I. multifiliis* in this study.

## 4. Discussion

Fluorescent live cell dyes have been widely used to analyze cell vitality and perform cell tracking. However, until now, there was no report about live cells of *I. multifiliis* labeled with fluorescent dye. In the preliminary study, the authors tried using CM-Dil (Yeasen, China) to label tomonts and theronts of *I. multifiliis*. However, CM-Dil could not permeate through the cysts of tomonts and the densely packed cilia on the surface of theronts of *I. multifiliis.* In contrast, CFDA-SE could permeate the plasma membrane and become strongly fluorescence retaining in living cells over 6 h (Figure 1). Although CFDA-SE is toxic to cells to a certain extent [21], there was no significant effect on the survival and motility of theronts of *I. multifiliis* under the concentration (1 μM) used in this study. 

In the life cycle of *I. multifiliis*, theronts are the infective stage to fish hosts. Therefore, killing or inhibiting theronts will prevent the invasion of fish which is essential to control white spot disease. Theronts, like most planktonic ciliates, are free-swimming in water. Although many studies revealed the top-down impact on ciliate community structure and biomass in the food webs by macro-zooplankton, especially cladocerans and copepods [22,23,24,25,26], until now, few reports have been published about the predation of copepods on *I. multifiliis*. However, most theronts, with a body size of 20–50 μm, fall within the prey size spectrum of macro-zooplankton. In the lab experiment of this study, the authors found that the live theronts labeled with fluorescence CFDA-SE were ingested by copepods, and strong fluorescent signals were observed in the alimentary canal (Figure 3). Furthermore, the challenge trials showed that the presence of zooplankton in the culture system could significantly mitigate the infection of *I. multifiliis* in goldfish (Figure 5). The results revealed that copepods could prey on the theronts of *I. multifiliis*. Therefore, eliminating theronts and disrupting the life cycle of *I. multifiliis* with copepods could be a potentially effective method for controlling white spot disease in aquaculture [2].

Planktonic ciliates are the main components of the microbial food web in both marine and freshwater ecosystems, and the abundance and species composition were significantly shaped by the different functional groups of macro-zooplankton predators [27]. Cladocerans, copepods and rotifers often co-occur in water and compete for limited food resources with different feeding behavior and efficiency. The feeding mechanism is strongly influenced by the availability of alternative food sources and by the motility, size and abundance of prey [28]. Most rotifers eat algal cells in the 4–17 μm range; most cladocerans are efficient filter feeders, ingesting suspended particles ranging in size from bacteria to algae and ciliates <30 μm [27]. In contrast, copepods selectively feed on larger prey and consume ciliates preferentially over alternative prey [29]. In this study, 11–18 species of copepods, cladocerans and rotifers were detected in the water samples; however, only mature copepods were found feeding on theronts of *I. multifiliis*. Meanwhile, one copepod nauplii individual was also found to be positive. Böttjer et al. [30] reported that *Oithona* spp. nauplii were important in controlling nanoplankton (3–20 μm) on the coast of Chile. Theronts of *I. multifiliis* are slightly larger in size, which may be difficult for nauplii to hunt. 

Copepods can be divided into filter feeding, predatory feeding and scraping feeding species. Some species of copepods, called mix-feeding types, can filter and be predatory. Food availability and body size are major factors shaping copepod feeding rates. Most Calanoids are filter feeding and lack the ability to hunt free-swimming theronts of *I. multifiliis*. Additionally, most harpacticoids crawl along the bottom of the water body; therefore, they were not detected in the water samples. Cyclopoid copepods are exclusively ambush feeding species that hunt relatively large and mostly motile prey [15,27]. In this study, we discovered seven Cyclopoid copepods that preyed on theronts. According to a previous report [15], *Macrocyclops albidus* and *Mesocyclops leuckarti* engaged in predatory feeding on insect larvae, oligochaeta, cladocerans and copepods; *Thermocyclops taihokuensis* was a fierce copepod feeding on fish eggs and cladocerans; *Microcyclops bicolor* was a mix-feeding copepod grazing on algae, protozoa, rotifers and animal carcasses. 

Notably, the cyclopoid *T. taihokuensis* comprised 62.5% of the copepods preying on theronts and were detected in all healthy ponds and lakes (NQ1, NQ2, NQ3 and LK1, LK2, LK3), but absent from samples collected from ponds where fish were heavily infected with white spot disease (YX1, YX2, YX3). Additionally, in all samples collected from NQ1, NQ2 and NQ3, *T. taihokuensis* was the only macro-zooplankton observed to eat fluorescence-labeled theronts. Therefore, we hypothesize that *T. taihokuensis* has a higher predation efficiency on theronts of *I. multifiliis*. 

In challenge tests, the results showed that the infection intensity of *I. multifiliis* in goldfish was significantly lower in the water with zooplankton. Dhanker et al. [28] found that the ciliate consumption rate of *Pseudodiaptomus annandalei* (Copepoda: Calanoida) was significantly lower in the presence of mixed algae. However, *Acanthocyclops robustus* (Copepoda: Cyclopoida) was found to be an effective biocontrol agent for eliminating the ciliate *Sterkiella* in cultures of the microalga *Chlamydomonas* without reducing microalgal production [31]. In this study, no significant difference (*p* > 0.01) was observed between the groups T+I and P+I or between the groups T+I+Z and P+I+Z, respectively. It suggests that zooplankton may preferentially feed on the theronts of *I. multifiliis* often over alternative prey, such as algae, smaller ciliates, in pond water. Furthermore, the results could well explain the phenomenon in aquaculture that fish in ponds with abundant plankton are less susceptible to white spot disease.

In conclusion, the present study determined the predation of cyclopoid copepods on theronts of *I. multifiliis*. The results indicate that seven species of copepods consumed fluorescence-labeled theronts, and the presence of zooplankton reduced the infection pressure of *I. multifiliis* on fish. This study uncovered a potential biological control method against white spot disease in aquaculture. However, further research is required to reveal the feeding efficacy of different copepod predators in the laboratory and field settings.

## Figures and Tables

**Table 1:** Information on macro-zooplankton samples collected in this study.

Sample Code	Type	Location	Health Condition of the Fish
NQ1-3	farming ponds	Nanquan Base of FFRC, Wuxi, Jiangsu	healthy
YX1-3	farming ponds	Nancheng Village, Yixing, Jiangsu	yellow-head catfish *Pelteobagrus fulvidraco* with serious white spot disease
LK1	wild water body	The Gonghu Lake, near Ping’an Bridge, Wuxi, Jiangsu	healthy
LK2	wild water body	The Taihu Lake, near Renzi Harbor Bridge, Wuxi, Jiangsu	healthy
LK3	wild water body	The Lihu Lake, near Changguangxi Bridge, Wuxi, Jiangsu	healthy

**Table 2:** Experimental grouping of zooplankton intervention on *I. multifiliis* infection.

Group Code	Water Source	Zooplankton	Theront (Cells)
T	2 L aerated tap water	null	null
T+I	2 L aerated tap water	null	50,000
T+I+Z	2 L aerated tap water	~1000 ind./L	50,000
P	2 L pond water	~1000 ind./L	null
P+I	2 L pond water	null	50,000
P+I+Z	2 L pond water	~1000 ind./L	50,000

**Table 3:** Copepod predators of theronts of *I. multifiliis* discovered in this study.

Species	Source	Numbers	Similar Species and Acc. No.		Sequence Similarity
*Paracyclopina* sp.	LK2–3	2	*P. nana*	FJ214952	90.62%
*Thermocyclops taihokuensis*	NQ1–3	60	*T. taihokuensis*	KR048801	99.22%
	LK1–3				
*Macrocyclops albidus*	LK2	1	*M. albidus*	KR048794	99.62%
*Macrocyclops* sp.	YX3	1	*M. distinctus*	KF153695	98.02%
			*Eucyclops roseus*	KR048792	96.03%
*Mesocyclops pehpeiensis*	LK2	2	*M. pehpeiensis*	KR048797	99.22%
*Mesocyclops* sp1.	YX1–3	27	*M. leuckarti*	KF153692	93.44%
			*M. pehpeiensis*	KR048797	93.05%
*Mesocyclops* sp2.	YX1–3	3	*M. leuckarti*	KF153692	89.96%

## Data Availability

The authors confirm that the data supporting the findings of this study are available within the manuscript and table.

## Supplementary Materials

The following supporting information can be downloaded at: https://www.mdpi.com/article/10.3390/pathogens12070860/s1, Table S1: Species composition of macro-zooplankton collected from fish ponds and lakes in this study.
