# Supplementary material for: Predation of Cyclopoid Copepods on the Theronts of Ichthyophthirius multifiliis: Shedding Light on Biocontrol of White Spot Disease
Source: Pathogens. 2023 Jun 22;12(7):860. doi: 10.3390/pathogens12070860 (PMC10386215; doi:10.3390/pathogens12070860)
Supplement: Supplementary file 1 [file pathogens-12-00860-s001.zip › pathogens-2459954-supplementary.pdf]

Table S1. Species composition of macro-zooplankton collected from fish ponds and lakes in this study.

Taxonomic group	Species	NQ1	NQ2	NQ3	YX1	YX2	YX3	LK1	LK2	LK3
	Asplachna brightwelli								+	
	Asplachna priodonta							+	+	+
	Asplachna sp.	+	+							
	Asplanchnopus multiceps			+						
	Brachionus angularis					+	+	+		+
	Brachionus budapestiensis			+			+			
	Brachionus calyciflorus	+	+	+	+		+	+	+	+
	Brachionus diversicornis	+	+	+	+	+			+	
	Brachionus sp.				+	+	+	+	+	
Rotifers	*Brachionus urceus*					+	+	+		
	Colurella sp.	+								
	Filinia longiseta					+	+			+
	Keratella cochlearis	+				+	+			+
	Keratella valaa			+	+	+	+		+	+
	Polyarthra euryptera									+
	Polyarthra trigla								+	+
	Pompholyx sulcate				+		+			
	Rotifera	+	+							
	Trichocerca porcsllus									+
	Bosmina longirostris					+	+	+	+	+
Cladocerans	*Bosminopsis deitersi*							+	+	
	Ceriodaphnia cornuta					+				
	Chydorus ovalis				+	+				

	Chydorus sphaericus				+		+			
	Daphnia sp.							+		
	Diaphanosoma brachyurum		+	+						
	Diaphanosoma leuchtenbergianum	+	+	+						
	Moina micrura	+	+	+		+				
	Sida crystallina							+		
	Simocephalus sp.							+		
	Copepod nauplii	+	+	+		+	+	+	+	+
	Copepodid	+	+	+	+	+	+	+	+	+
	Diaptomidae			+						
	Eucyclops serrulatus		+	+				+		
	Eucyclops macrurus			+						
	Schmackeria forbest					+	+			
	Sinocalanus dorrii			+	+	+				
Copepods	*Macrocyclops* sp.			+			+		+	+
	Mesocyclops sp1.				+	+	+			
	Mesocyclops sp2.				+	+	+			
	Mesocyclops pehpeiesis								+	+
	Microcyclops sp.					+				
	Microcyclops longiramus							+		
	Paracyclopina sp.								+	+
	Thermocyclops taihokuensis	+	+	+				+	+	+
Total		11	11	16	11	18	17	15	15	16

Note：This is the result of qualitative identification, "+" only represents the species identified, not the quantity.
